# From Striatum to Prescription: An Evidence-Based and Bayesian Framework for Neuromotor Rehabilitation in Parkinson’s Disease

**DOI:** 10.3390/neurosci7050101

**Published:** 2026-09-05

**Authors:** Alessandro Rossi, Federica Ginanneschi

**Affiliations:** 1 Rehabilitative Research Foundation Gianfranco Salvini ETS, 52025 Montevarchi, Italy; 2 Department of Medical, Surgical and Neurological Sciences, University of Siena, 53100 Siena, Italy

**Keywords:** Bayesian ranking, cortical compensation, multimodal rehabilitative interventions, neuroplasticity, striatal recalibration, Parkinson’s disease

## Abstract

Parkinson’s disease (PD) involves progressive basal ganglia dysfunction, with hyperexcitability of striatal indirect-pathway D2 medium spiny neurons (D2-MSNs) linked to motor impairment. Neuromotor rehabilitation is an important therapy, but its efficacy varies across interventions. This thematic review examines eleven rehabilitative strategies for PD, spanning forced and voluntary exercise (FE and VE respectively), non-invasive brain stimulation (rTMS, tDCS), and technology-based approaches such as exoskeletons, augmented reality, and dual-task training, within a framework distinguishing striatal recalibration from compensation via alternative motor networks. To formalize this distinction, a Bayesian ranking framework combines neurobiological plausibility with clinical evidence quality, identifying three functional clusters: a high-recalibration cluster (FE, *p* ≈ 0.80; LSVT BIG, *p* ≈ 0.62), an intermediate-uncertainty cluster (rTMS, HIIT, tDCS, treadmill, resistance training, Tai Chi/dance; 0.40–0.56), and a bypass/compensatory cluster (augmented reality, exoskeletons, dual-task training; *p* ≤ 0.33). This distinction between direct modulation of basal ganglia circuitry and recruitment of alternative motor networks, including the lateral premotor cortex, parieto-premotor circuits, and cerebello-thalamo-cortical pathways, supports a precision rehabilitation approach in PD.

## 1. Introduction

Parkinson’s disease (PD) affects more than ten million people worldwide and is projected to double in prevalence by 2040, making it the fastest-growing neurological pandemic of the contemporary era [1]. Progressive loss of dopaminergic neurons in the substantia nigra pars compacta and the resulting nigrostriatal insufficiency produce the classical motor triad, bradykinesia, rigidity, and resting tremor, compounded by postural instability, freezing of gait (FoG), and a rich nonmotor symptom profile. Pharmacological therapy with levodopa and dopamine agonists remains the cornerstone of symptomatic management, but does not alter the neurodegenerative trajectory and progressively loses efficacy as the disease advances.

In this context, neuromotor rehabilitation has acquired over the past two decades a role that transcends simple functional compensation, emerging as a potentially neuromodulating and, in some animal models, neuroprotective intervention. The cellular mechanisms underlying these effects have been progressively elucidated. Converging evidence, ranging from the modulation of striatal D2 receptor expression by high-intensity treadmill exercise in the MPTP mouse model [2,3,4] to electrophysiological findings showing that aerobic exercise reduces beta-band power and D2-MSN firing frequency [5], has culminated in the causal evidence provided by Chen et al. [6] through chemogenetic inhibition and re-excitation of D2 medium spiny neurons (D2-MSNs). Their findings confirm a mechanism supported by nearly two decades of converging evidence. However, not all exercise is equivalent: the distinction between voluntary exercise (VE) and forced or supervised exercise (FE) emerges as the critical variable that determines access to these striatal recalibration mechanisms. Concurrently, a second therapeutic axis has emerged from neuroimaging research: parieto-premotor and cerebellar compensation, long regarded merely as a mere epiphenomenon of basal ganglia dysfunction, is now recognised as an active and potentiable mechanism in its own right. Johansson et al. [7] demonstrated that interindividual variability in motor symptom severity is determined by the degree of parieto-premotor compensation rather than by the extent of striatal dopaminergic loss; longitudinal data further indicate that the rate of bradykinesia progression tracks the decline of cortical compensation, not the progression of nigrostriatal dysfunction [8]. Non-invasive brain stimulation and technologies-based rehabilitation approaches —exoskeletons, augmented reality (AR), and multimodal cueing systems—operate precisely on this second axis, offering tools to amplify and direct compensatory plasticity. The present paper proposes that these two axes—striatal recalibration and cortical compensation—are not alternative but complementary targets of a precision rehabilitation strategy, and presents a Bayesian framework for integrating the evidence supporting each approach. This is a thematic review, organised around this specific mechanistic question, and does not aim to provide a comprehensive or systematic account of all rehabilitative interventions available for PD. Its contribution lies not in cataloguing existing interventions more exhaustively than prior syntheses, but in reorganising heterogeneous evidence around the distinction between striatal recalibration and alternative-pathway compensation, and in formalising this distinction through an explicitly Bayesian, updatable framework.

### 1.1. Main Review Question

This thematic review addresses the integration of exercise and emerging neuromotor rehabilitation strategies to target striatal recalibration and cortical compensation in PD, and examines how these complementary mechanisms may inform precision rehabilitation.

### 1.2. Secondary Objectives

To address this question, the review aims to achieve the following: 1. Examine the neurobiological basis of motor dysfunction in PD, with particular emphasis on the cellular and circuit mechanisms underlying striatal dysfunction and their progressive elucidation through preclinical and clinical evidence. 2. Critically evaluate exercise-based rehabilitation, including lower- and upper-limb exercise, with particular focus on the distinction between VE and FE, and their respective clinical and neurobiological effects. 3. Assess the evidence for exercise-induced striatal recalibration, including the potential role of upper-limb exercise as a model of systemic corticostriatal modulation. 4. Examine cortical compensation as a complementary therapeutic target, including the contribution of parieto-premotor and cerebellar networks to motor function and disease progression. 5. Review non-invasive brain stimulation and emerging rehabilitation technologies, including exoskeletons, augmented reality, and multimodal cueing systems, as approaches to enhance compensatory cortical plasticity. 6. Propose a Bayesian integrative framework for precision rehabilitation, aimed at combining evidence from the two therapeutic axes while explicitly acknowledging the different levels and sources of evidence supporting striatal recalibration and cortical compensation.

The literature search was conducted using PubMed, Pedro, Scopus, and Web of Science and covered the period from 1980 to the present. Search terms included combinations of terms related to the main mechanistic and clinical themes addressed in the review, including “Parkinson’s disease”, rehabilitation”, “exercise”, “forced exercise”, “cadence”, “basal ganglia”, “D2-MSN”, “striatum”, “neuroplasticity,” “motor circuitry”, and “compensation.” The search was complemented by screening the reference lists of relevant articles and reviews. Consistent with the thematic nature of the review, the literature was not selected according to a predefined systematic-review protocol. Rather, studies were considered on the basis of their relevance to the mechanistic questions addressed, methodological quality, and their contribution to the principal lines of evidence discussed in the review.

## 2. Cellular Substrate of Motor Dysfunction and Its Modifiability

Motor control by the basal ganglia depends on the balance between two parallel pathways: the direct pathway (D1 medium spiny neurons [D1-MSNs]), which facilitates movement by inhibiting the internal globus pallidus (GPi) and substantia nigra pars reticulata (SNr), and the indirect pathway (D2 medium spiny neurons [D2-MSNs]), which inhibits movement by activating the sub-thalamic nucleus and thereby GPi/SNr. Under physiological conditions, dopamine exerts opposing effects on the two pathways, exciting the direct pathway via D1 receptors and inhibiting the indirect pathway via D2 receptors. This dynamic balance enables the selection of desired motor programmes while suppressing competing ones [9,10].

In PD, dopaminergic depletion releases D2-MSNs from inhibitory modulation, generating pathological hyperexcitability of the indirect pathway, the so-called “neural brake”, which non-selectively suppresses motor output and generates bradykinesia, rigidity, and difficulty initiating movement. This D2-MSN hyperactivity is not merely a correlate of pathology but its principal mechanistic driver within the dopaminergic striatal system, as demonstrated with causal rigour by Chen et al. [6] in a 6-hydroxydopamine (6-OHDA) mouse model: chemogenetic inhibition of D2-MSNs fully recapitulated the motor benefit of treadmill exercise, while chemogenetic re-excitation of the same population abolished exercise-induced recovery.

Preclinical evidence from the past several years converges in indicating that physical exercise acts on this hyperexcitability through multiple molecular mechanisms: increased brain-derived neurotrophic factor (BDNF) expression and tropomyosin receptor kinase B (TrkB) receptor activation with subsequent Erk1/2 phosphorylation and cAMP response element-binding protein (CREB) activation in the nigrostriatum [11,12]; modulation of glial cell line-derived neurotrophic factor (GDNF) expression with neuroprotective effects on residual nigrostriatal projections [13]; reduction in striatal neuroinflammation through down-regulation of CD11b/c (integrin alpha-M/integrin alpha-X) in macrophages and Iba1 in microglia [14]; and reduction in alpha-synuclein oligomerisation [15]. Dopaminergic plasticity induced by voluntary exercise requires, however, intact striatal BDNF as a necessary and sufficient condition [16]. This finding is specific to the VE paradigm and evidence reported in [16] and should not be extended to exercise-induced plasticity more broadly, including that induced by FE.

## 3. Exercise in Parkinson’s Disease: Lower and Upper Limbs

### 3.1. Lower Limbs: The Voluntary Versus Forced Distinction

The distinction between VE and FE was initially investigated by Ridgel et al. [17]. In their study, patients with mild-to-moderate PD underwent eight weeks of assisted cycling at a cadence 30% above their preferred voluntary rate (FE) or at their self-selected cadence (VE). The FE group showed a 35% reduction in Unified Parkinson’s Disease Rating Scale (UPDRS) motor scores and improved bimanual dexterity, whereas the VE group did not show comparable motor benefits, despite achieving higher power output and heart rate. These findings provided initial clinical evidence that externally imposed cadence may confer benefits beyond those attributable to voluntary exercise intensity alone.

Alberts et al. [18] subsequently proposed that the effects of FE may be related not simply to metabolic demand, but also to the frequency and density of proprioceptive afferent input generated by the externally imposed cadence. Inputs from muscle spindles, Golgi tendon organs, and joint mechanoreceptors may increase sensorimotor and corticostriatal activity and thereby contribute to activity-dependent plasticity. Preclinical evidence further suggests that exercise-related modulation of D2-expressing medium spiny neurons (D2-MSNs) may contribute to motor improvement in dopamine-depleted striatal circuits [6]. However, the extent to which this mechanism directly mediates the clinical effects of FE in humans remains uncertain.

The 30% increase above the preferred voluntary cadence used by Ridgel et al. [17] has therefore been adopted as an operational characteristic of the original FE paradigm. Importantly, current evidence does not establish 30% as a biological threshold required to induce neuroplasticity, nor does it demonstrate that smaller increases in cadence are ineffective. Rather, it represents the experimentally tested cadence difference associated with clinical benefit in the original study. Whether a specific minimum increase above voluntary cadence is necessary to reproduce the neurobiological and clinical effects attributed to FE remains an open question.

### 3.2. Upper Limbs: The Axial Declension of the Forced Paradigm

Forced exercise is not conceptually bound to cycling or the lower limbs. Any modality that imposes on the central nervous system a frequency of activation or a recruitment pattern superior to the patient’s voluntary capacity constitutes FE. Applied to the upper limbs and cervico-scapular girdle via biomechanically constraining devices, this paradigm generates high-density proprioceptive afference from cervical and scapular districts, areas with broad sensorimotor cortical representation, constituting a corticostriatal driver topographically distinct from lower-limb FE with potential to modulate postural and vestibular circuits not accessible via cycling alone [19].

Messa et al. [19] demonstrated, via functional magnetic resonance imaging (fMRI) with Arterial Spin Labelling and resting-state connectivity analysis, that 16 sessions of supervised upper-limb exercise with a specially designed mechanical device (in which the seated patient performed repeated shoulder abduction and adduction movements against a progressive load, with movement frequency and range biomechanically constrained beyond the patient’s voluntary capacity) produced significant increases in cerebral blood flow in the primary motor cortex (M1), supplementary motor area, and cerebellar cortex, alongside corticostriato-cerebellar connectivity changes, with improvements in UPDRS-III and dynamic posturography extending to districts not directly trained. Ginanneschi et al. [20] replicated and extended these findings in a two-arm randomised controlled trial (RCT). The vestibular ratio of the Sensory Organisation Test, which quantifies the capacity to rely on vestibular input for postural stability when visual and somatosensory cues are simultaneously absent or unreliable, improved significantly only in the supervised arm, while the somatosensory and visual SOT ratios remained unchanged in both groups. The same supervised protocol produced no measurable benefit in age-matched healthy controls. This dissociation cannot be explained by peripheral muscular or proprioceptive adaptations, which would be expected to occur equally in healthy subjects. Instead, it points to a disease-specific central recalibration that operates only in the presence of pathological indirect-pathway overactivity amenable to correction. The convergent clinical and neuroimaging findings reported here, comprising cerebral blood flow changes, connectivity, UPDRS, and posturography, are compatible with disease-specific central recalibration but do not themselves constitute direct proof of D2-MSN involvement in humans.

### 3.3. Critical Appraisal

Strengths: FE is the rehabilitative intervention in PD with the most robust neurobiological rationale and translational coherence across levels of analysis, including cellular [6], neuroimaging, and clinical evidence [17,19,20]. The lower- and upper-limb paradigms are complementary: lower-limb FE accesses the sensorimotor circuits of gait, whereas upper-limb FE engages postural and vestibular networks, with evidence of systemic cross-modal recalibration absent in healthy controls. The approximately +30% cadence increment is operationally meaningful within the classic forced-exercise paradigm but may be difficult to sustain without supervision. Thus, the supervised/forced paradigm represents the condition under which these effects have, to date, been empirically documented. Whether smaller cadence increments or unsupervised variants of the paradigm can produce comparable clinical and neurobiological effects remains an open question that cannot be resolved by the current evidence.

Methodological limitations: Samples rarely exceed 20–30 patients per arm and follow-up rarely exceeds 8–12 weeks. All patients were on stable dopaminergic therapy under standardised on-medication assessment conditions, which reduces but does not eliminate the pharmacological confound. A second transversal bias is disease duration heterogeneity: samples are rarely stratified by degree of nigrostriatal depletion, which directly determines the D2-MSN substrate available for recalibration and therefore the expected magnitude of FE response.

Open questions: Whether the D2-MSNs recruited by lower- and upper-limb FE are localised within distinct striatal territories; whether the therapeutic efficacy of FE is limited to a specific stage of the neurodegenerative process; and whether FE and levodopa interact in an additive, synergistic, or independent manner to promote striatal recalibration.

## 4. Non-Invasive Brain Stimulation: rTMS and tDCS

Non-invasive brain stimulation fits into the framework of this review as a top-down modulator of the corticostriatal pathway: whereas FE acts bottom-up through proprioceptive afference, rTMS and tDCS act from above by modifying cortical excitability and the connectivity of corticostriatal projections. The rationale is convergent, but the strength of evidence is asymmetric, and this asymmetry deserves to be stated frankly.

### 4.1. Repetitive Transcranial Magnetic Stimulation (rTMS)

High-frequency rTMS on M1 and the supplementary motor area (SMA) represents the neurostimulation intervention with the largest number of randomised controlled trials (RCTs) in PD. The increase in corticomotor excitability induced by high-frequency stimulation (5–25 Hz) reduces the suppression of motor output mediated by the hyperactive indirect pathway, acting complementarily to FE but via the descending pathway. Recent meta-analyses [21,22] confirm significant effects on bradykinesia and rigidity, with UPDRS-III improvements in the range of 10–20%. The multifocal bilateral approach with H-coil (Deep TMS), which simultaneously stimulates M1 and the prefrontal cortex (PFC), is the only one to address in a single session both the motor and cognitive-executive components of PD [23]. Cerebellar rTMS represents an emerging area: the cerebellum, through the dentato-thalamo-cortical circuit, can indirectly modulate corticostriatal connectivity with promising but not yet consolidated effects on balance and gait. These effects should be read alongside the substantial protocol heterogeneity and limited durability discussed below, which qualify the strength of the evidence summarised here.

#### Critical Appraisal of rTMS

Strengths: rTMS has the largest evidence base among non-invasive neurostimulation techniques for PD, a neurobiological rationale consistent with the indirect-pathway model, and a multifocal approach with the potential to improve both motor and cognitive symptoms simultaneously.

Methodological limitations: The effect sizes are modest and interindividual variability is high. The heterogeneity of stimulation parameters across studies (including frequency, intensity, number of trains, target, and cycle duration) precludes the identification of an optimal protocol and limits the feasibility of direct comparisons. A convincing sham remains methodologically difficult. The duration of effects generally does not exceed 4–8 weeks from the end of the cycle, with relevant implications for clinical sustainability. It remains unclear whether rTMS acts predominantly by recalibrating the corticostriatal pathway or by compensating via alternative pathways, the same conceptual distinction that discriminates FE from cueing.

Open questions: Adequately powered RCTs directly comparing rTMS and FE, or testing their sequential combination (rTMS as “priming” of cortical plasticity before FE), are lacking. The interaction with dopaminergic therapy, in particular the optimal temporal window relative to levodopa administration, has not been systematically studied.

### 4.2. Transcranial Direct Current Stimulation (tDCS)

Anodal tDCS on M1 increases cortical excitability and normalises dysfunctional cortical connectivity in PD through long-term potentiation (LTP)-like synaptic potentiation mechanisms. Compared to rTMS it offers relevant practical advantages—low cost, portability, and the possibility of simultaneous administration during exercise without interfering with training—a characteristic that makes it the natural candidate for combined protocols. The meta-analysis by Giustiniani et al. [24] documents its effects on cognitive as well as motor outcomes, with implications for quality of life in patients with frontal involvement. The trial by Pisano et al. [25] combining cerebellar tDCS with an augmented reality treadmill produced results superior to those of the individual components, validating the principle of neurostimulation–exercise synergy.

#### Critical Appraisal of tDCS and Combined Approaches

Strengths: tDCS’ portability and low cost open the prospect of home neuromodulation; there is the unique possibility of stimulation simultaneous with exercise; cognitive effects have been documented beyond motor ones; and there is initial evidence of the superiority of the tDCS + exercise combination over those components individually.

Methodological limitations: Effect sizes on motor symptoms are on average lower than those of rTMS and spatial specificity of continuous current is limited. Electrode montage is often chosen empirically rather than on a neuronavigated basis. Duration of post-cycle effects is brief (2–4 weeks). A proportion of subjects correctly identify the active condition from cutaneous sensation, with potential blinding compromise. Interindividual response variability, influenced by skull thickness, composition of underlying tissue, and baseline excitability state, is large and not predictable.

Open questions: The optimal dose–response profile is not defined. Timing relative to exercise—before, during, or after—has not been systematically compared. Whether cerebellar tDCS offers specific advantages on axial symptoms over M1 stimulation has no answer yet from adequately powered RCTs.

## 5. Emerging Rehabilitative Technologies: Between Bypass and Recalibration

The technologies described in this section—exoskeletons, AR, multimodal cueing systems, and dual-task training—share a characteristic that fundamentally distinguishes them from FE and brain stimulation: they do not act on the root of the problem (D2-MSN hyperexcitability and the consequent motor output suppression), but compensate for its dysfunctional output by activating alternative motor pathways. This distinction, recalibration versus bypass, is not a criticism but a conceptual clarification that must guide the clinical integration of these tools: they are complementary to FE, not substitutes.

### 5.1. Exoskeletons and Gait Assistance Devices

Wearable exoskeletons correct the pathological characteristics of the gait cycle—reduced step length, hypokinesia, asymmetry, and freezing—through active mechanical assistance. Powered lower-limb exoskeletons are wearable battery-operated devices that impose a normative locomotor pattern on the patient’s gait cycle, constraining step length, cadence, and joint kinematics beyond what the patient can generate voluntarily, thereby functioning, at least in part, as a form of lower-limb forced exercise rather than purely passive mechanical assistance. Randomised trials in PD patients have documented significant improvements in gait parameters and postural stability with this class of devices. [26]. The soft robotics device developed by Kim et al. [27], which assists hip flexion in synchrony with the gait cycle, significantly reduced freezing in ecological conditions, configuring a paradigm that imposes on the central nervous system a temporal activation pattern that the patient cannot generate autonomously, with conceptual analogies to FE. Assistive and rehabilitative processes are, in this sense, not mutually exclusive from the outset: exoskeletons may facilitate movement while simultaneously promoting task-specific practice, motor learning, and longer-term neural adaptation.

#### Critical Appraisal of Exoskeletons

Strengths: Among the rehabilitative technologies considered in this review, exoskeletons are, to our knowledge, the only ones capable of active motor assistance in ecological conditions even in patients with severe motor deficits; soft robotics overcomes the weight and bulk limitations of traditional rigid exoskeletons.

Methodological limitations: Clinical trials in PD are still numerically limited, and the devices tested are heterogeneous. High cost also limits accessibility outside research settings. A fundamental question—whether the benefits reflect lasting plastic carry-over, acute assistance, or a combination of both—has not been systematically addressed in PD. The key unresolved issue is therefore to determine to what extent exoskeleton training induces benefits that persist beyond device use and whether these effects are associated with the neural mechanisms proposed for FE, including D2-MSN recalibration, rather than representing assistance that is predominantly dependent on the device. Clarifying this distinction will be essential for understanding the long-term rehabilitative potential of exoskeleton-based interventions in PD.

Open questions: RCTs explicitly comparing exoskeleton training versus conventional FE on neuroimaging and striatal biomarker outcomes are needed to clarify whether the two paradigms access the same plasticity mechanisms or distinct pathways.

### 5.2. Augmented Reality and Multimodal Cueing Systems

Freezing of gait responds poorly to both pharmacological therapy and traditional physiotherapy. External cueing—visual, acoustic, tactile, or multimodal—exploits the relatively preserved capacity to use external signals in PD to compensate for the dysfunction of internal automatic motor programmes, engaging the lateral premotor cortex and dorsolateral visuomotor pathways that bypass the basal ganglia. Augmented reality delivers contextualised visual cues in real environments, overcoming the limitations of immersive virtual reality (VR) in terms of safety and detachment from the daily environment. Hoogendoorn et al. [28] documented significant modifications of gait kinematic parameters with AR cueing. The ELIMINATE FoG trial [29] compared different AR cueing strategies, and the pragmatic RCT CAPARE trial [30] (completed February 2025) tested gamified AR gait and balance rehabilitation at home, opening the prospect of ecological home neurorehabiliation.

#### Critical Appraisal of Augmented Reality and Multimodal Cueing Systems

Strengths: These systems have a favourable risk–benefit profile; are applicable in real and home environments; and achieve multimodal cueing adaptable to individual patient parameters. The home AR paradigm extends rehabilitation to the ecological daily space.

Methodological limitations: The effect of cueing is acute and situational: it decays rapidly upon signal removal, with limited evidence of long-term transfer to no-cue conditions. This follows from how the bypass paradigm is defined in this review: by construction, it does not modify the pathological substrate, but temporarily circumvents it. Interindividual variability in response to cueing is marked: some patients respond better to visual, others to acoustic cueing, and FoG itself is neurobiologically heterogeneous (cholinergic vs. dopaminergic vs. mixed), making uniform response unlikely. AR trial samples are still limited and outcome measures heterogeneous. There is also the theoretical risk that prolonged dependence on external cueing reduces the stimulus for reorganisation of internal motor programmes, a paradoxical effect that warrants systematic study.

Open questions: It remains unclear whether prolonged AR training can induce lasting plastic changes in lateral premotor pathways, transforming a compensatory bypass mechanism into genuine recalibration, or whether the effects remain limited to the period of active use. The combination of FE and AR-based cueing, which could simultaneously target striatal recalibration and premotor bypass mechanisms, has not yet been investigated as an integrated intervention, despite theoretically representing one of the most comprehensive strategies currently available.

### 5.3. Dual-Task Training and Virtual Environment

PD markedly impairs the ability to perform dual tasks, such as walking while talking, carrying objects, or responding to external stimuli, reflecting reduced attentional resources available for automatic gait control. Dual-task training delivered through VR or AR provides a safe, controlled environment in which this ability can be systematically trained with progressively increasing task complexity. The systematic review by Lin et al. [15] suggests that dual-task training is superior to single-task training in improving combined motor and cognitive outcomes, with benefits that are consistent with the reorganisation of frontoparietal attentional–motor networks.

#### Critical Appraisal of Dual-Task Training

Strengths: Dual-task training has strong ecological validity, as most falls in people with PD occur during dual-task situations, and evidence suggests its superiority over single-task training in improving combined motor and cognitive outcomes.

Methodological limitations: The definition of “dual-task” varies considerably across studies in terms of the type, difficulty, and modality of presentation of the secondary task. Furthermore, study samples systematically exclude patients with significant cognitive impairment, who represent the most vulnerable population and may potentially derive the greatest benefit from this intervention. The specific contribution of the VR/AR component versus flat-screen dual-task training has not been systematically compared.

Open questions: Transfer of effects from the training setting to untrained real-world daily conditions remains insufficiently documented. Moreover, the optimal integration of dual-task training with physical exercise and brain stimulation as components of a structured multimodal rehabilitation protocol has not yet been systematically investigated.

### 5.4. Additional Rehabilitative Procedures: HIIT, Treadmill Training, Tai Chi/Dance, Resistance Training, and LSVT BIG

The most extensive comparative synthesis available, a Cochrane network meta-analysis [31] including more than 150 RCTs and 7000 participants, identified aerobic exercise and balance/gait training as the modalities associated with the largest effect sizes on UPDRS-III scores and functional mobility, while mind–body approaches demonstrated comparable benefits for postural stability. Notably, FE, as defined in this review, is not represented as a distinct category within this synthesis; its trials are subsumed under broader classifications such as aerobic or cycling exercise. This absence is itself methodologically significant, given the specific neurobiological rationale and mechanistic evidence discussed above.

High-intensity interval training (HIIT): HIIT is distinguished from FE by the absence of external biomechanical constraint: it acts on the dopaminergic pathway and neuroplasticity predominantly through BDNF release and neurotrophic factors linked to metabolic intensity, rather than through proprioceptive recalibration of the striatal indirect pathway. The meta-analysis by Harpham et al. [32] documents significant effects on UPDRS-III and aerobic capacity, with adequate feasibility and safety. HIIT therefore represents a procedure mechanistically distinct from FE, complementary for clinical effects, with a neuroprotective rationale, mediated by BDNF and reduction in alpha-synuclein oligomerisation, and more documented than FE but with less demonstrated effects on striatal recalibration.

Treadmill training: Conventional treadmill training, distinct from forced-rate treadmill training because it does not necessarily impose a fixed cadence, is the intervention with the most extensive body of RCT evidence in PD for gait outcomes. The meta-analysis by Bishnoi et al. [33], which included 13 RCTs, demonstrated significant improvements in UPDRS-III, gait velocity, and the Timed Up and Go test (TUG) compared with other gait-training modalities. However, the effect appears to be speed-dependent: studies using self-selected speeds showed smaller effect sizes than those imposing progressive increases in speed. This pattern is compatible with, although insufficient to establish, the forcing component as a critical variable, since exercise intensity, training volume, and patient characteristics were not systematically disentangled across the included trials.

Tai Chi and dance: Tai Chi has the most solid evidence base among mind–body procedures in PD: the meta-analysis by Lou et al. [34] documents significant improvements in balance (BBS), functional mobility (TUG), and gait velocity versus conventional pharmacological therapy. The rationale—combination of postural control, focused attention, and rhythmic coordination—activates sensorimotor and attentive–frontal circuits distinct from those of the striatal indirect pathway, configuring a mechanism of bypass and reinforcement of alternative pathways rather than D2-MSN recalibration. Dance (particularly the Argentine tango) shows analogous effects, with an additional social and musical component, but with more limited evidence in terms of study numbers.

Resistance training: The meta-analysis of 15 RCTs by Song et al. [35] documents significant improvements in UPDRS-III and gait velocity both for aerobic training and for resistance training. The latter acts on rigidity and muscle strength through peripheral mechanisms (hypertrophy, motor unit recruitment) and central mechanisms (corticomotor plasticity modulation), with striatal-pathway effects less documented than in FE but consistent effect sizes for primary motor symptoms.

LSVT BIG: The Lee Silverman Voice Treatment BIG (LSVT BIG) programme is an intensive amplitude-based training protocol designed to address reduced movement amplitude and improve the scaling of motor output in PD. Clinical studies have reported improvements in motor performance and functional outcomes, although the certainty of evidence for some patient-reported outcomes, including quality of life, remains limited [31]. From a mechanistic perspective, LSVT BIG explicitly targets the perception and scaling of movement amplitude through intensive, cognitively guided practice. This provides a biologically plausible route for enhancing sensorimotor integration and motor learning, but direct evidence that LSVT BIG induces specific changes in basal ganglia circuit activity or D2-MSN function in humans remains limited. Thus, its clinical efficacy, proposed mechanism, and biological plausibility should be considered as distinct levels of evidence. Recent feasibility and safety data [36] further support its applicability in hospital settings as an alternative to intensive treadmill-based training.

#### Comparative Critical Appraisal

Strengths of the overall landscape: The availability of an updated Cochrane network meta-analysis [31] directly comparing 13 exercise modalities on common outcomes represents a rare methodological base in neurological rehabilitation. Convergent evidence indicates that structured physical exercise, regardless of modality, is superior to inactive control for PD motor symptoms, with aerobic and balance/gait training in a relative position of advantage.

Transversal limitations: Direct cellular evidence for striatal recalibration is currently limited or unavailable for most of the procedures described in this section. This represents an important gap in the evidence rather than evidence that such mechanisms do not occur. The pharmacological treatment and disease duration biases discussed apply fully to these studies as well. Heterogeneity of protocols within each category, intensity, duration, frequency, and setting produces effect size estimates with wide confidence intervals and often high I^2^, limiting the clinical translatability of recommendations.

Open questions: Direct comparisons between FE and HIIT, between forced and self-selected treadmill speeds, and between LSVT BIG and upper-limb FE on neuroimaging outcomes are lacking. These comparisons are necessary to build a prescription hierarchy based on mechanism rather than clinical habit.

## 6. Bayesian Ranking of Procedures: A Framework for Precision Prescription

One of the principal weaknesses of narrative reviews in rehabilitation is the lack of a formal framework that transparently integrates evidence of heterogeneous quality with an explicit neurobiological prior. Here, we propose the application of a Bayesian ranking approach as a tool to formalise the evaluative process conducted in the preceding sections, thereby making it reproducible, updatable, and falsifiable.

The logic of the framework can be summarised as follows. Before examining the clinical evidence, we assign to each rehabilitative procedure a prior probability, an initial estimate of how likely it is that the procedure acts through recalibration of striatal D2-MSN hyperexcitability, derived exclusively from the neurobiological evidence reviewed in Section 1, Section 2, Section 3, Section 4 and Section 5. This prior reflects the knowledge derived from cell biology and translational neuroscience that is available before examining the findings of RCTs: FE receives a high prior because its proposed mechanism is supported by convergent evidence at the cellular level; AR cueing receives a low prior because it is explicitly designed to bypass rather than recalibrate the defective pathway. FE is treated here as one intervention among the eleven considered, evaluated under the same criteria as the others; the strength of its prior reflects the strength of its supporting mechanistic evidence, and does not constitute a reference standard against which the other interventions are validated. We then update this estimate in light of the available RCTs: procedures supported by multiple positive trials move their probability upward; those with few or inconsistent trials move it less, but retain the credit of a strong neurobiological prior. The result, the posterior probability, is a single number between 0 and 1 that integrates both sources of knowledge. Crucially, a low posterior probability does not mean a procedure is clinically useless: it means its therapeutic benefit is more likely to flow through compensatory pathways than through direct striatal recalibration. The width of the uncertainty band around each estimate (the 95% credibility interval) reflects how much epistemic room remains: wide intervals signal that more RCT evidence is needed; narrow intervals signal relative confidence. The framework is designed to be updated iteratively as new trials are published, making it a living synthesis rather than a fixed ranking.

### 6.1. The Formal Model

For each rehabilitative procedure i, we define R_i_ as the event “the procedure produces genuine striatal D2-MSN recalibration” (distinct from bypass/compensation). This distinction is introduced here as a mechanistic hypothesis to be evaluated against the evidence, not as an established classification of these procedures. The posterior probability of this event, given the available clinical evidence E_i_, is determined by Bayes’ theorem:

P(R_i_|E_i_) = [P(E_i_|R_i_)·P(R_i_)]/P(E_i_) (1)

where P(R_i_) is the prior on the recalibration mechanism, derived from cell biology and translational coherence [6,13]; P(E_i_|R_i_) is the likelihood of the observed clinical evidence given that mechanism; and P(R_i_|E_i_) is the posterior probability of D2-MSN recalibration.

### 6.2. Prior Specification

The prior P(R_i_) is modelled as a Beta(α, β) distribution, the natural conjugate distribution for probabilities in [0, 1]. The parameters α and β are calibrated on the neurobiological rationale: α is high if direct cellular evidence of D2-MSN modulation exists (FE), and α is low if the mechanism is predominantly bypass (AR cueing). The prior parameters for each procedure, justified by the biology discussed in the preceding sections, are as follows: Upper- and lower-limb FE: Beta(7,2), strong prior, mechanism causally demonstrated [6]. LSVT BIG: Beta(6,3), recalibration of motor amplitude via cognitive–attentional pathway. rTMS: Beta(5,5), top-down corticostriatal modulation; plausible mechanism but not demonstrated at the cellular level. tDCS: Beta(4,5), convergent rationale but limited spatial specificity. HIIT: Beta(5,4), effects on BDNF and neuroprotection; D2-MSN modulation less documented. Resistance training, treadmill, Tai Chi/dance: Beta(3,5-6), predominantly peripheral and bypass mechanisms. Dual-task/VR-AR, exoskeletons, AR cueing: Beta(2,6-8), bypass paradigm by definition. For the procedures assigned lower priors, this reflects, as noted in Section 5.4, an absence of direct cellular evidence rather than a demonstrated reliance on bypass mechanisms alone.

#### Sensitivity Analysis

To assess whether the reported clustering depends critically on the specific prior parameters chosen, we conducted a sensitivity analysis varying, for each procedure, the prior concentration (halving and doubling α + β while holding the prior mean fixed) and the prior mean (±20%, holding concentration fixed), recomputing the posterior mean under each perturbation. This analysis leaves the framework’s two most consequential claims unaffected: the high-recalibration classification of forced exercise, whose posterior mean remained above 0.70 across the entire range tested, and the bypass classification of AR cueing, exoskeletons, and dual-task training, all of which remained stable throughout. Four procedures, LSVT BIG, rTMS, HIIT, and Tai Chi/dance, whose baseline posterior means lie close to the boundaries separating the three clusters, showed sufficient sensitivity to prior calibration to shift their nominal cluster label under moderate perturbation. In no case, however, did any of these four procedures move toward the opposite extreme: none approached the bypass cluster’s range, nor did any settle stably within the high-recalibration range. Their movement was confined to the region of mechanistic ambiguity already implied by their classification as an intermediate cluster in this review, and did not influence the framework’s central architecture.

### 6.3. Likelihood and Bayesian Updating

The likelihood P(E_i_|R_i_) is modelled as a binomial likelihood: for each procedure, the number of available RCTs producing positive and replicable results (s, successes) out of the total (*n*), weighted for methodological quality (Cochrane risk of bias), is counted. A trial was counted as positive if it reported a prespecified primary outcome improvement of clinical or statistical significance in the expected direction, and as replicable when this result was corroborated by at least one independent trial where available. The resulting posterior distribution is analytical:

P(R_i_|E_i_) ∼ Beta(α + s, β + f) (2)

where f = *n* − s is the number of failures. The posterior mean is (α + s)/(α + β + *n*) and the 95% credibility interval (highest density interval, HDI) reflects residual epistemic uncertainty: biologically grounded priors with few RCTs produce wide intervals; weak priors with many RCTs produce narrower intervals. The model is updatable iteratively with each newly published trial, rendering it a living evidence synthesis tool.

### 6.4. Results and Interpretation

**Figure 1:**
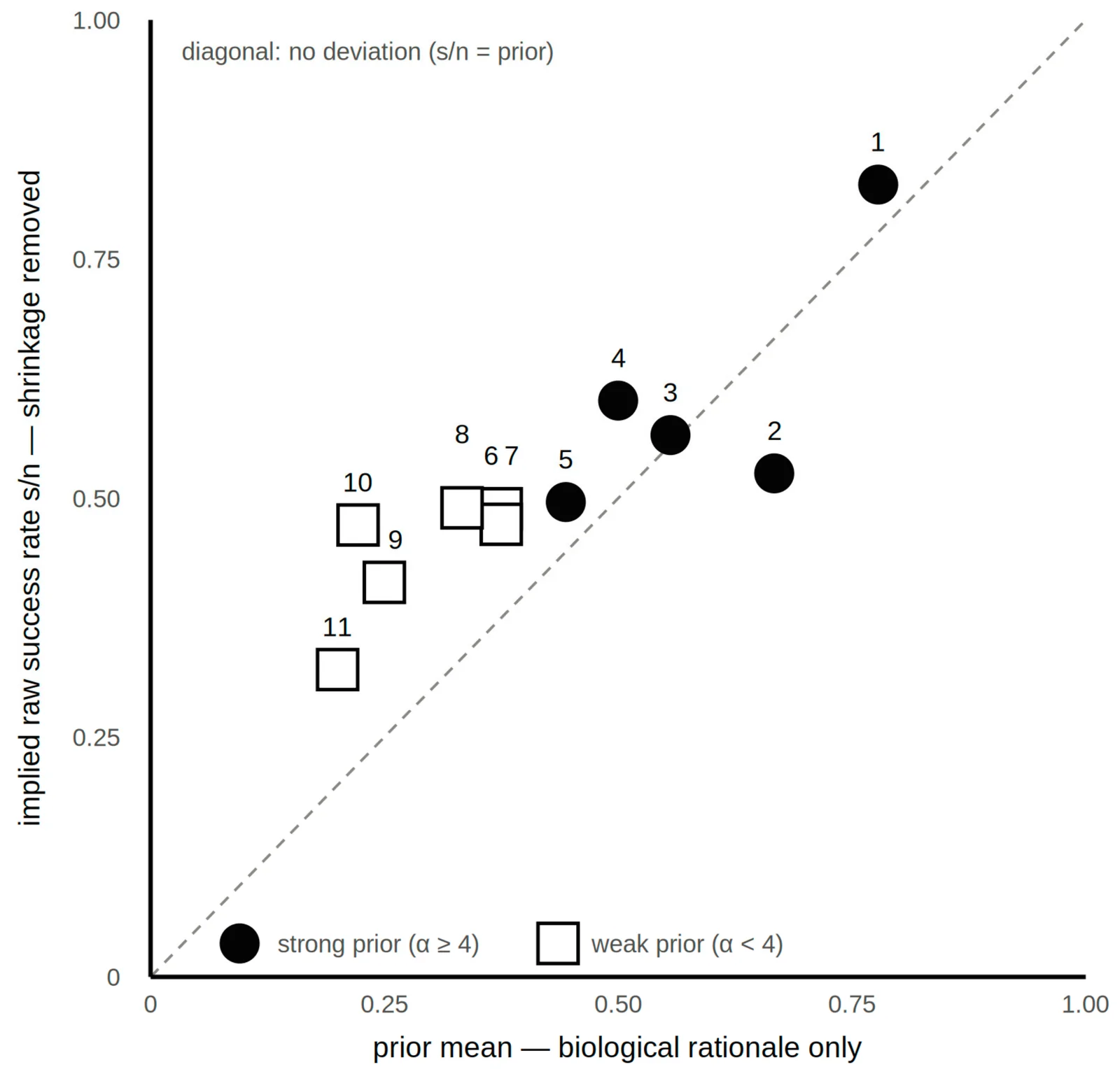
Prior mean versus implied raw clinical evidence, with shrinkage removed. In Beta-Binomial updating, shrinkage refers to the systematic pull of the posterior mean toward the prior mean whenever the clinical data (*n* RCTs) carry less statistical weight than the prior (α + β): P(R|E) = (α + s)/(α + β + *n*), so the posterior is mechanically constrained to remain close to the prior whenever *n* is small, regardless of how strong or weak the underlying clinical signal actually is. For each procedure, the x-axis reports the prior mean α/(α + β), derived exclusively from the neurobiological rationale (Section 6.2). To bypass this shrinkage, the y-axis does not report the posterior mean P(R|E) itself, but the underlying empirical success rate s/*n* implied by the clinical evidence, algebraically recovered from the posterior and prior Beta parameters as *n* = (α′ + β′) − (α + β) and s = α′ − α, where α′ and β′ are the parameters of the posterior distribution estimated by moment-matching to the reported posterior mean and 95% HDI (Section 6.4). Plotting s/*n* directly against the prior mean isolates the genuine clinical signal from the shrinkage constraint. The dashed diagonal represents no deviation between the raw clinical evidence and the prior expectation. Filled circles indicate procedures with a strong prior (α ≥ 4); open squares indicate a weak prior (α < 4). Points 6 and 7 (resistance training and treadmill training) are drawn as two overlapping squares because the manuscript reports an identical prior and posterior mean for both; an explicit 95% HDI is given only for treadmill training and was used as an approximation for resistance training, for which the text does not report a 95% HDI. The recovered *n* and s values are themselves algebraic reconstructions from the reported summary statistics, not the original trial counts used by the authors, and should be read as indicative rather than exact. 1 = forced exercise; 2 = LSVT BIG; 3 = HIIT; 4 = rTMS; 5 = tDCS; 6 = resistance training; 7 = treadmill training; 8 = Tai Chi/dance; 9 = dual-task/VR-AR; 10 = exoskeletons; 11 = AR cueing.

Figure 1 illustrates the relationship between prior and posterior estimates after accounting for the shrinkage inherent to the Beta-Binomial model when the available clinical evidence is limited. Removing the shrinkage effect provides a complementary view of the extent to which procedure-specific clinical evidence departs from the corresponding neurobiological prior. LSVT BIG (point 2) shows a relatively marked negative deviation from the diagonal, suggesting that the available clinical evidence is less supportive than would be expected from its assigned prior. This finding is consistent with the Cochrane classification of the evidence for quality-of-life outcomes as “very uncertain” (Section 6.4). Conversely, dual-task/VR-AR (point 9) and AR cueing (point 11) show relatively large positive deviations, indicating that the available clinical evidence is more favourable than suggested by their assigned mechanistic priors. In particular, the findings for AR cueing are consistent with the acute clinical improvements in freezing of gait reported in Section 5.2. These deviations should nevertheless be interpreted cautiously, as they are influenced by both the underlying clinical evidence and the assumptions incorporated into the priors and model. Thus, Figure 1 should be regarded as a sensitivity-oriented visualisation of the relationship between clinical evidence and mechanistic expectations, rather than as a direct measure of the strength of either component.

**Figure 2:**
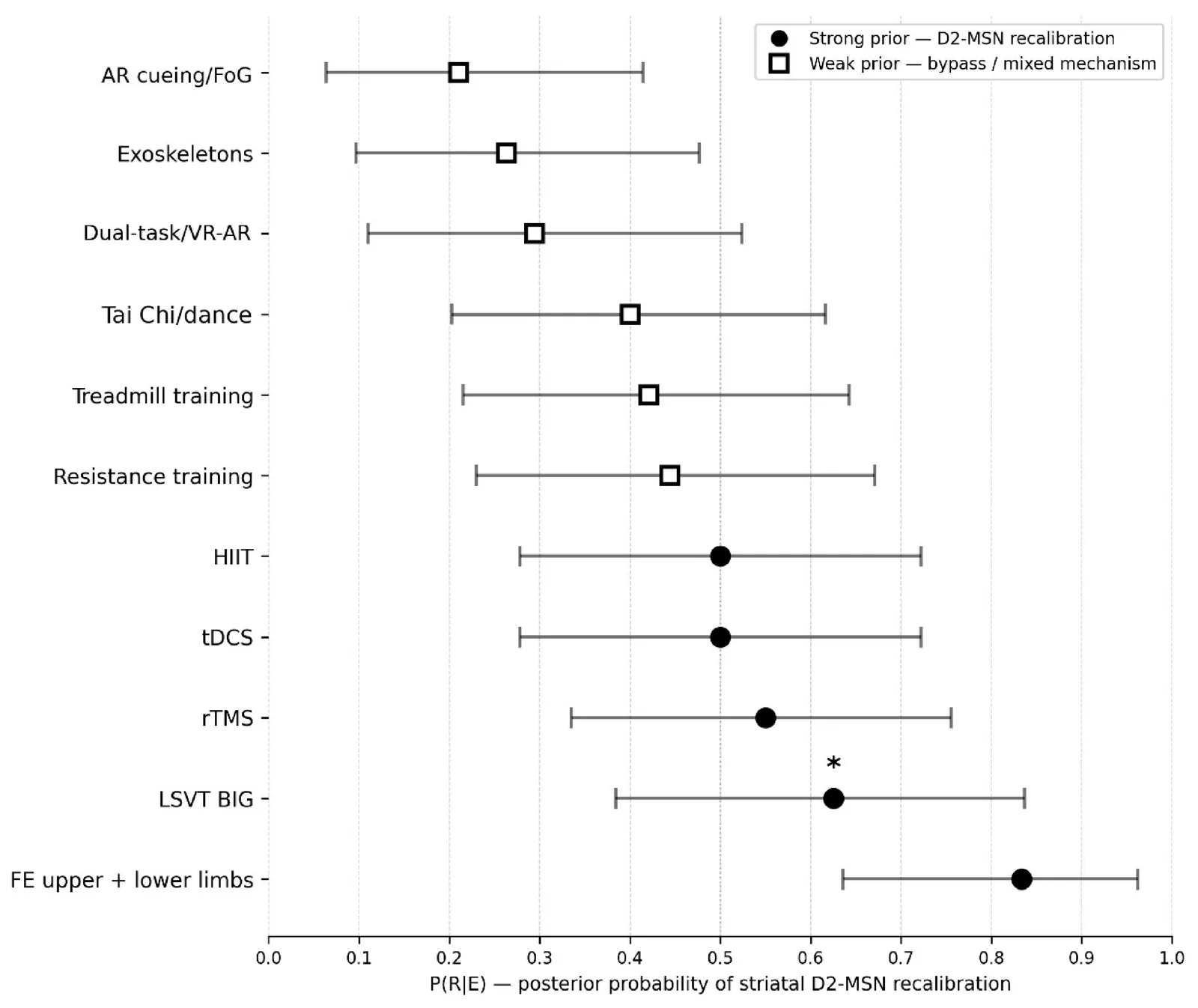
Bayesian ranking of rehabilitative procedures in Parkinson’s disease. For each procedure, the posterior probability P(R|E) of striatal D2-MSN recalibration (x-axis) is reported, estimated via a Beta-Binomial model with a prior calibrated on the neurobiological mechanism and a likelihood derived from available RCTs. Horizontal bars represent 95% credibility intervals (HDI, highest density interval). Filled circles indicate procedures with a strong prior (α ≥ 4), i.e., with a neurobiologically grounded D2-MSN recalibration rationale; open squares indicate procedures with a weak prior (α < 4), whose predominant mechanism is bypass or compensation. The vertical dotted line at P = 0.5 separates procedures with higher posterior probability (recalibration likely) from those with lower probability (bypass likely). * LSVT BIG: Once the Beta-Binomial shrinkage is removed (Figure 1), the implied raw clinical evidence falls below this procedure’s prior expectation, the only such case among the eleven procedures—i.e., the neurobiological rationale currently promises more than the available evidence has shown, consistent with the Cochrane classification of LSVT BIG evidence on quality of life as “very uncertain” (Section 6.4). Methodological note: P(R|E) is not a measure of overall clinical efficacy, but specifically of the probability that the procedure’s mechanism of action passes through recalibration of striatal D2-MSN hyperexcitability, as distinct from the bypass of alternative motor pathways. A procedure with low P(R|E) may be clinically effective through compensation mechanisms (see text). FE = forced exercise; HDI = highest density interval; LSVT BIG = Lee Silverman Voice Treatment BIG; rTMS = repetitive transcranial magnetic stimulation; tDCS = transcranial direct current stimulation; HIIT = high-intensity interval training; AR = augmented reality; FoG = freezing of gait.

Figure 2 reports a Bayesian ranking of rehabilitative procedures in PD. For each procedure, the posterior probability P(R_i_|E_i_) of striatal D2-MSN recalibration is reported, estimated using a Beta-Binomial model with procedure-specific priors. The procedures are presented along a continuous gradient of estimated recalibration probability. Upper- and lower-limb FE show the highest posterior estimates, followed by LSVT BIG, whereas rTMS, HIIT, tDCS, resistance training, treadmill training, and Tai Chi/dance occupy an intermediate range. Dual-task/VR-AR training, exoskeletons, and AR cueing show lower posterior estimates. These differences should be interpreted as differences in the estimated probability of the proposed mechanism within the specified Bayesian model, rather than as rankings of overall clinical efficacy.

The relatively high estimate for FE reflects the combination of its assigned prior and available mechanistic and clinical evidence, including preclinical evidence implicating D2-MSN activity [6] and convergent neuroimaging and motor findings [17,18,19,20]. The posterior estimate for LSVT BIG is also influenced by its relatively strong prior despite a more limited RCT evidence base. Conversely, the wider HDIs observed for several interventions reflect substantial uncertainty arising from the available evidence and model assumptions. For interventions with lower posterior estimates, including dual-task/VR-AR training, exoskeletons, and AR cueing, the results should not be interpreted as evidence against clinical efficacy or as proof that their effects are mediated exclusively by compensatory pathways. Rather, they indicate that the available evidence provides less support, within the present model, for D2-MSN-mediated recalibration.

**Figure 3:**
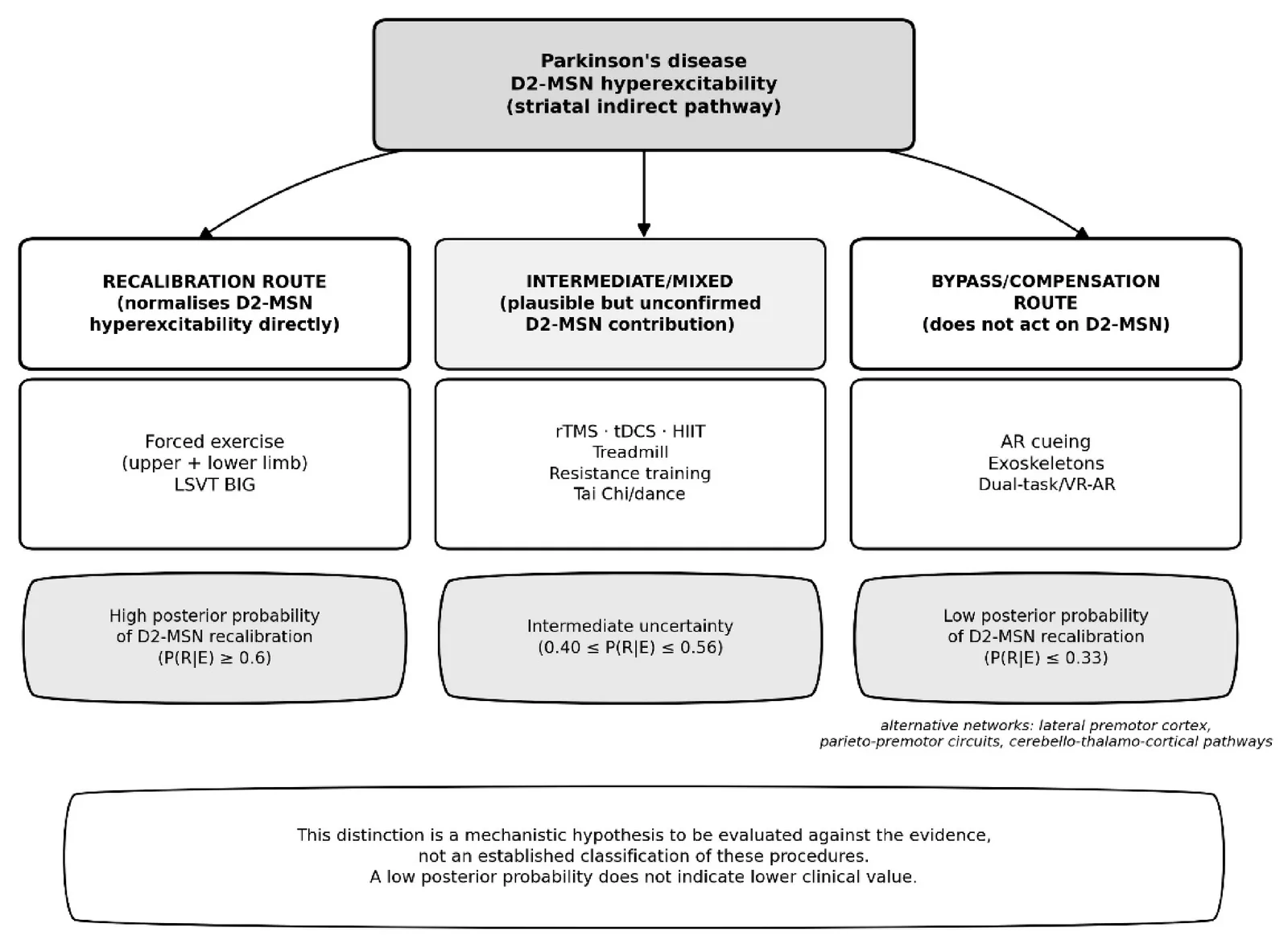
Conceptual framework: Striatal recalibration versus alternative-pathway compensation. The eleven procedures are grouped according to the three posterior-probability clusters reported in Figure 2. The recalibration and bypass routes are shown as the two mechanistic axes introduced in Section 1; the intermediate column corresponds to procedures for which the available evidence does not yet discriminate between the two mechanisms. As throughout this review, the three-way grouping shown here is offered as a testable mechanistic distinction rather than a fixed taxonomy, and a lower posterior probability of D2-MSN recalibration carries no implication of reduced clinical value.

The vertical line at P = 0.50 should therefore be interpreted as a descriptive reference point within the Bayesian framework rather than as a biological or efficacy threshold. As established in Section 6.1, this distinction remains a hypothesis to be tested rather than a settled classification. Further experimental studies, particularly those directly assessing neural and cellular mechanisms in humans, are required to determine whether the proposed mechanistic distinctions correspond to distinct biological pathways. In Figure 3, the eleven procedures are grouped according to the three posterior-probability clusters reported in Figure 2.

## 7. Towards an Integrative Model: Precision Exercise Prescription in PD

Before articulating clinical implications derivable from the evidence discussed, it is necessary to acknowledge two systematic biases that run transversally through the entire rehabilitative literature in PD and that condition the strength of any inference. The first is the pharmacological confound: all studies enrol patients on dopaminergic therapy, often with non-standardised doses and clinical evaluations not systematically conducted in the off-medication state. Since levodopa and dopamine agonists modify corticomotor excitability, corticostriatal connectivity, and D2 receptor sensitivity, the effect of the rehabilitative intervention is never measurable in isolation from its pharmacological substrate. The second is the disease duration bias: studies mix patients with very different neurodegenerative trajectories, ignoring that the degree of residual dopaminergic depletion, and therefore the substrate on which striatal recalibration can operate, varies in a non-linear fashion with disease progression. Future research cannot do without designs that explicitly stratify by disease duration, Hoehn & Yahr stage, and medication state at the time of assessment.

The evidence discussed in this thematic review is compatible with a proposed interpretive model that may guide rehabilitative prescription in PD. At the centre of this proposed model lies the pathological hyperexcitability of the striatal indirect pathway (D2-MSN), understood, as elsewhere in this review, within the dopaminergic striatal system; its plausible extension to postural, sensorimotor, or vestibular deficits beyond this system remains to be demonstrated and is not assumed here. The most effective rehabilitative interventions are those that normalise this hyperexcitability (FE, supervised exercise) or that compensate for its dysfunctional output by activating alternative pathways (AR cueing, dual-task training).

This model generates testable predictions and clinical implications:

First, available evidence suggests that modality matters more than intensity. Here, “modality” refers specifically to the voluntary/forced distinction, not to exercise intensity as conventionally measured, e.g., in metabolic or cardiovascular terms; the comparison is not intended more broadly. FE must exceed by 30% or more (that is, the cadence increment adopted in the classic FE paradigm) the patient’s voluntary cadence, and exercise must be supervised or biomechanically constrained. Self-selected voluntary physical activity, while beneficial for cardiovascular and metabolic health, has not been shown to engage the striatal recalibration mechanisms proposed to underlie the effects observed with FE.

Second, body segment matters, and the constraint applies to upper limbs too. FE is not confined to cycling: its application to the upper limbs and cervico-scapular girdle, via devices that impose a frequency and recruitment pattern superior to the voluntary threshold, generates proprioceptive afference from the districts most compromised in PD and most extensively represented in sensorimotor cortex. This axial FE is the only one to produce the cross-modal recalibration documented by Ginanneschi et al. [20], including the vestibular benefit absent in healthy controls, and must be conceptually distinguished from merely supervised or assisted upper-limb exercise.

Third, brain stimulation can amplify training but not substitute for it. rTMS and tDCS on M1 modulate cortical excitability and corticostriatal connectivity, but their effect is maximised when combined with exercise that provides the afferent activity substrate on which stimulation operates.

Fourth, digital technologies can extend rehabilitation to ecological space. Exoskeletons, AR, and multimodal cueing systems do not replace but complement clinical training, enabling generalisation of learned motor patterns to everyday environments.

Fifth, the healthy vs. patient response functions as a biomarker. The absence of benefit in healthy controls subjected to the same supervised protocols [20] suggests that the response to FE is a candidate physiological signature of indirect-pathway dysfunction, pending prospective validation, an observation that could be exploited to personalise rehabilitative prescription as a function of individual patients’ neurophysiological phenotypes.

## 8. Conclusions

Rehabilitation in PD may be undergoing a profound conceptual transformation: the evidence discussed in this review is compatible with a shift from a purely symptomatic-compensatory intervention towards a potentially disease-modifying neuroplastic strategy, grounded in identifiable cellular and circuit mechanisms potentially exploitable with precision. The distinction between voluntary and forced/supervised exercise is not a technical subtlety, but the variable that separates a generically healthful activity from an intervention that is hypothesised to reconfigure the dynamics of the striatal indirect pathway, the cellular substrate of motor deficits in PD.

The evidence from Messa et al. [19] and Ginanneschi et al. [20] and the preclinical findings of Chen et al. [6] provide complementary evidence: the cellular mechanism demonstrated in mice provides a rationale for the systemic effects observed in humans, while the pathology-dependent selectivity of the clinical effects supports the translational relevance of the animal model. However, the present findings in humans do not directly demonstrate D2-MSN involvement and should therefore be interpreted as consistent with, rather than confirmatory of, the mechanism proposed on the basis of animal studies. Non-invasive brain stimulation and emerging digital technologies are complementary tools in this framework: the former amplify the window of plasticity top-down, while the latter extend the rehabilitative influence bottom-up to real daily-life environments.

The Bayesian ranking framework proposed in this review provides, for the first time in this field, a formal and reproducible tool for integrating neurobiological priors with clinical evidence quality, making explicit the epistemic asymmetry between interventions hypothesised to promote recalibration of the pathological substrate and those that primarily compensate for its functional consequences through alternative pathways. This distinction represents the principal conceptual contribution of the present work and may help guide the design of future precision-exercise RCTs in PD.

The challenge for future research is threefold: to identify the neurophysiological biomarkers (sub-thalamic local field potentials (LFPs), corticostriatal functional magnetic resonance imaging connectivity, gait kinematic parameters) that predict individual patients’ responses to different interventions; to test in adequately powered RCTs the combined exercise–brain stimulation–digital technology protocols that the integrative model proposed in this review identifies as the most promising frontier of precision rehabilitation in PD; and, most critically, to design the first head-to-head trials comparing procedures of recalibration and bypass on the same patients, with washout periods long enough to distinguish durable plasticity from transient compensation.

Open questions: Recalibration versus bypass remains a key issue. No RCT has yet directly compared a recalibration procedure (FE, LSVT BIG) with a bypass procedure (AR cueing, dual-task training) on the same patients, with adequate washout periods and neuroimaging endpoints. The available evidence is nevertheless coherent with the predictions of the model in three ways. First, durability: Cueing effects decline rapidly upon removal of the external signal, as demonstrated by the RESCUE trial [37], in which no carry-over to functional outcomes was observed six weeks after cessation of cueing training, a pattern consistent with bypass dependency. By contrast, supervised high-intensity exercise modifies the clinical trajectory over years when maintained as a maintenance programme [38]. Second, there is a partial exception: Nieuwboer et al. [37] reported that nine sessions of cueing produced limited improvements even under uncued conditions and hypothesised that prolonged cueing may re-route movement through lateral premotor pathways, precisely the bypass-to-recalibration transition that this review identifies as an open question. Third, the absence of direct comparisons is itself informative: the recalibration/bypass distinction proposed here creates a testable question that the existing literature has not yet formulated. The critical experiment would compare, in a parallel-group RCT with neuroimaging endpoints and a minimum 12-week washout, forced exercise against AR cueing matched for total session time, measuring both acute and durable effects on UPDRS-III, gait kinematics, and corticostriatal fMRI connectivity. Such a trial would provide the first empirical test of the framework’s central prediction: that recalibration procedures produce larger, more durable effects on striatal biomarkers, while bypass procedures produce larger acute effects on freezing and gait in advanced disease stages where the D2-MSN substrate is depleted.

## Data Availability

Data are available on request from the corresponding author.

## Abbreviations

The following abbreviations are used in this manuscript:

6-OHDA, 6-hydroxydopamine; AR, augmented reality; BBS, Berg Balance Scale; BDNF, brain-derived neurotrophic factor; BIG, see LSVT BIG; CD11b/c, integrin alpha-M/integrin alpha-X; CREB, cAMP response element-binding protein; D1-MSNs, D1 medium spiny neurons; D2-MSNs, D2 medium spiny neurons; FE, forced exercise; fMRI, functional magnetic resonance imaging; FoG, freezing of gait; GBD, Global Burden of Disease; GDNF, glial cell line-derived neurotrophic factor; GPi, internal globus pallidus; HDI, highest density interval; HIIT, high-intensity interval training; LFP, local field potential; LTP, long-term potentiation; LSVT BIG, Lee Silverman Voice Treatment BIG; M1, primary motor cortex (area); MAPK, mitogen-activated protein kinase; PD, Parkinson’s disease; PFC, prefrontal cortex; RCT, randomised controlled trial; RCTs, randomised controlled trials; rTMS, repetitive transcranial magnetic stimulation; SMA, supplementary motor area; SNr, substantia nigra pars reticulata; SOT, Sensory Organisation Test; tDCS, transcranial direct current stimulation; TrkB, tropomyosin receptor kinase B; TUG, Timed Up and Go test; UPDRS, Unified Parkinson’s Disease Rating Scale; VE, voluntary exercise; VR, virtual reality.
